# Studying Early Lethality of 45,XO (Turner's Syndrome) Embryos Using Human Embryonic Stem Cells

**DOI:** 10.1371/journal.pone.0004175

**Published:** 2009-01-12

**Authors:** Achia Urbach, Nissim Benvenisty

**Affiliations:** Department of Genetics, Silberman Institute of Life Sciences, The Hebrew University, Jerusalem, Israel; Brunel University, United Kingdom

## Abstract

Turner's syndrome (caused by monosomy of chromosome X) is one of the most common chromosomal abnormalities in females. Although 3% of all pregnancies start with XO embryos, 99% of these pregnancies terminate spontaneously during the first trimester. The common genetic explanation for the early lethality of monosomy X embryos, as well as the phenotype of surviving individuals is haploinsufficiency of pseudoautosomal genes on the X chromosome. Another possible mechanism is null expression of imprinted genes on the X chromosome due to the loss of the expressed allele. In contrast to humans, XO mice are viable, and fertile. Thus, neither cells from patients nor mouse models can be used in order to study the cause of early lethality in XO embryos. Human embryonic stem cells (HESCs) can differentiate in culture into cells from the three embryonic germ layers as well as into extraembryonic cells. These cells have been shown to have great value in modeling human developmental genetic disorders. In order to study the reasons for the early lethality of 45,XO embryos we have isolated HESCs that have spontaneously lost one of their sex chromosomes. To examine the possibility that imprinted genes on the X chromosome play a role in the phenotype of XO embryos, we have identified genes that were no longer expressed in the mutant cells. None of these genes showed a monoallelic expression in XX cells, implying that imprinting is not playing a major role in the phenotype of XO embryos. To suggest an explanation for the embryonic lethality caused by monosomy X, we have differentiated the XO HESCs *in vitro* an *in vivo*. DNA microarray analysis of the differentiated cells enabled us to compare the expression of tissue specific genes in XO and XX cells. The tissue that showed the most significant differences between the clones was the placenta. Many placental genes are expressed at much higher levels in XX cells in compare to XO cells. Thus, we suggest that abnormal placental differentiation as a result of haploinsufficiency of X-linked pseudoautosomal genes causes the early lethality in XO human embryos.

## Introduction

Turner's Syndrome results from X chromosome monosomy. This syndrome described in 1938 [1], is the most common sex chromosome abnormality in females [2]. Females with Turner's syndrome have several characteristic phenotypes, the most common are growth failure, gonadal dysgenesis and webbed neck [2], [3]. 3% of all pregnancies start with XO embryos, however it is estimated that only 1% of these embryos survive to term [2], [4]. Moreover, among the Turner's syndrome patients more than 50% are mosaic (e.g. 45,X0/46,XX) [2] which may suggest that the frequency of survival of 45,XO fetuses is even less than 1%. Most of the miscarriages of XO embryos occur during the first trimester [4].

Several molecular mechanisms have been suggested to explain the phenotypes observed in Turner's syndrome [5]. Of these mechanisms the most plausible explanation is haploinsufficiency of genes that are normally expressed from the two X chromosomes. In this case, the affected phenotypes are the result of the dosage of the specific gene. Another logical explanation is the presence of imprinted genes on the X chromosome, which are expressed in a mono-allelic fashion. In this case, the loss of the expressed chromosome will result in null expression of the gene. The underlying assumption of these two proposed mechanisms is that the involved genes must escape X inactivation. Otherwise, according to the imprinting hypothesis there will be no expression of the genes in half of the cells upon X inactivation, and according to the haploinsufficiency hypothesis all the cells will not have sufficient dosage of the gene upon X inactivation. In addition, according to the haploinsufficiency hypothesis the gene must have an active homolog on the Y chromosome, so that the expression levels in males will be equal to those in females. One major argument against the imprinting hypothesis is that it predicts that the phenotype will depend upon the parental origin of the single X chromosome and there is very little evidence for this [5].

Based on the haploinsufficiency assumption, 3 candidate genes have been proposed in order to explain the etiology of Turner's syndrome. Fisher et al. [6] has isolated the RPS4X (NM_001007) and its homolog RPS4Y (NM_001007) genes. Another gene that has been suggested to have a role in Turner's syndrome phenotypes is the ZFX gene [7]. However others have argued that haploinsufficiency in these genes are not the cause of Turner's syndrome [5], [8].

The only gene that has been proven to be associated with one of the phenotypes of Turner's syndrome is SHOX (NM_000451). This gene was isolated by two groups in parallel [9], [10] by positional cloning of an area in pesudoautosomal region 1 (PAR1) which was deleted in individual with short stature.

One major disadvantage in previous studies of Turner's syndrome is that all of the patients represent the exceptional case (1%) that survived to term. Thus no conclusion about the most prevalent phenotype of the 45,XO karyotype, namely the early lethality, can be reached by studying these patients.

It has been shown that XO mice, in contrast to humans, are viable and are anatomically normal and fertile [11]. Hence XO mice can not be used as a model for Turner's syndrome and for the embryonic lethality caused in monosomy of the X chromosome.

Human embryonic stem cells (HESCs) can differentiate in culture into structures called embryoid bodies [12]. In these EBs one can detect cells from the three embryonic germ layers as well as extra-embryonic cells. Moreover, genes that characterize gastrulation and organogenesis in mammals are temporally regulated during differentiation of HESCs into EBs [13], [14]. In addition, HESCs can differentiate into the three embryonic germ layers *in vivo* when injected into SCID mice [15]. Thus, these pluripotent cells have a great value in studying human developmental genetic diseases, particularly in cases where the mouse model fails to recapitulate the phenotypes seen in the patients. Previously, we have utilized HESCs in order to study two genetic diseases. We created a model of Lesch-Nyhan syndrome by gene targeting of the HPRT1 gene (NM_000194) [16], and a model of Fragile X syndrome by deriving a new HESC line from an affected blastocyst identified by preimplantation genetic diagnosis (PGD) to carry fragile X syndrome [17].

In our current research we have used HESCs in order to study the cause of miscarriage in XO embryos. Since most of the XO embryos die during the first trimester [4], HESCs, that differentiate into various embryonic cells, can be the best source of cells for studying the reasons for the early lethality in XO embryos. We have thus isolated HESCs that have spontaneously lost an X chromosome. By analyzing the gene expression profile of the cells upon differentiation we have searched for affected tissues and for new candidate genes to explain the lethality in 45,XO human embryos.

We suggest that loss of the X chromosome causes a defect in differentiation of HESCs into the trophectoderm placenta.

## Materials and Methods

### Cell Culture

HESC lines H9, HUES9, and I3 were cultured as previously described [12], [18]. Wild-type HESCs were transfected with pEGFP-N1 plasmid (Clontech) using the calcium phosphate method as described previously [19]. Stably transfected clones were established by neomycin selection (0.1 µg/ml, Sigma) following transfection. Some of the clones have a t(1;17) translocation which appear also in the WT cells.

Undifferentiated cells were trypsinized and induced to form EBs by allowing them to aggregate in suspension culture by growing them in nonadherent plastic petri bacterial dishes in the absence of bFGF as previously described [12], [18]. EBs were collected for analysis following 30 days of cell aggregation in culture.

### Induction of Teratomas

All animal experiments were conducted under the supervision of the Hebrew University Faculty of Sciences Animal Care and Use Committee (license NS-01-05). Teratomas were formed by injection of 1–5×10^6^ ES cells, under the kidney capsule of SCID/beige mice. Teratomas were isolated 5–8 weeks following injection.

### RNA extraction and RT-PCR analysis

RNA was extracted using Total RNA Extraction kit (RBC) or TRI-reagent (Sigma) for total RNA isolation according to the manufacturer's instructions. cDNA was synthesized using random hexamer primers. Amplification was performed using RedTaq ReadyMix PCR reaction mix (Sigma) or FastStart Taq DNA polymerase (Roche) for products longer than 1 kb. PCR conditions for most of the reaction include a first step of 3 minutes or 6 minutes (for cDNA and gDNA respectively) at 94°C, a second step of 35 cycles of 30 seconds at 94°C, 30 seconds at 60°C, 1–3 minute at 72°C (depened on the product's length) and a final step of 10 minutes at 72°C.

The conditions for the Amelogenin genes were: 94°C for 5 min and than 35 cycles of 94°C for 45 sec 55°C for 45 sec and 72°C for 1 min and then a final step of 10 minutes at 72°C

Primers are listed in supplementary Table S3. Final products were examined by gel electrophoresis on 1–2% agarose ethidium bromide-stained gels.

qRTPCR were carried out using either SYBER Green ROX Mix (ABgene) or TaqMan probes (Applied Biosystems) with TaqMan universal master mix (Roche), according to the manufacturer's instructions using the 7300 Real-Time PCR System (Applied Biosystems). Some of qRTPCR reactions were performed using the TaqMan® Human Stem Cell Pluripotency Array (4385344) according to the manufacturer's instructions. For description of primers see supplementary Tables S4, S5.

### FISH analysis

Cells were fixed with methanol and acetic acid (3∶1). Fluorescent in situ hybridization was carried out according to the manufacturer's recommendations and as described previously [20] using probe mixtures specific for chromosomes X, Y and 17 [Vysis, Downers Grove, IL] The criterion for signal scoring was that signals had to be at a minimum of a signal's width apart to be scored as two separate signals. Confocal imaging was performed using an MRC-1024 Bio-Rad confocal scan head coupled to a Zeiss Axiovert 135 M inverted microscope (Carl Zeiss, Jena, Germany).

### Microsatellite DNA Analysis

Genomic DNA was extracted from culture cells using EZ-DNA kit (Biological Industries, Kibbutz Beit Haemek, Israel) according to the manufacturer's instructions. Genotyping was performed at the Center for Genomic Technologies at the Hebrew University of Jerusalem, Israel, using the dxs1106 and dxs1060 markers from the Genethon human linkage map (ABI PRISM Linkage Mapping Set MD10; Applied BioSystems, Foster City, CA). Polymerase chain reaction (PCR) amplification of individual markers (fluorescence-dye-labeled forward primer and unlabeled reverse primer) was performed in a PTC 225 DNA Engine (Bio-Rad, Hercules, CA) using 25–30 ng of genomic DNA, 6 pmoles of each primer, 1.5 mM MgCl2, 0.14 mM deoxynucleoside-5-triphosphate, 1× PCR Gold Buffer and 0.4 units of AmpliTaq Gold DNA Polymerase (both from Applied Biosystems) in a total volume of 10 µl. PCR conditions were as follows: an initial 12 minutes denaturation at 95°C, 10 cycles of 15 seconds at 94°C, 15 seconds at 55°C and 30 seconds at 72°C, 10 minutes at 72°C, and hold forever at 10°C. After amplification, 1–2 µl were sampled into 9 µl of loading buffer (formamide with GeneScan-400HD [ROX] size standard; Applied Biosystems). PCR product electrophoresis and detection were performed using the 3700 Automated DNA Analyzer (Applied Biosystems). Sizing and genotyping were performed using GENSCAN and GENOTYPER software (Applied Biosystems).

### DNA Microarray Analysis

Total RNA was extracted according to the manufacturer's protocol (Affymetrix, Santa Clara, CA, http://www.affymetrix.com) from populations of undifferentiated, *in vitro* and *in vivo* differentiated cells derived from normal and XO HESCs. Hybridization to the U133A microarrays, washing, and scanning were performed according to the manufacturer's protocol (Affymetrix), and expression patterns were compared between samples. Signals were normalized by dividing each probe in the average value of the DNA microarray and scaled to a mean value of 100, to avoid differences between different DNA microarrays and experiments. Probes were then floored to a value of 20 to avoid superficially high ratios in respect to non expressed probes. In each case, two samples of XO clones were compared to three samples of normal cells. Volcano plots were generated using Partek software (Partek Inc. St. Louis, MO.; http//:www.partek.com).

### SNP analysis

DNA and cDNA were subjected to PCR and RT-PCR, respectively, using the primers listed in supplementary Table S6. The PCR products was purified and subjected to fluorescent dideoxy sequencing. In order to avoid DNA contamination in the RT-PCR, the primers for the RT-PCR were chosen from different exons. In cases that the PCR products were longer than 2 kb nested RT-PCR was performed

## Results

### Isolation of 45,XO HESCs clones

**Figure 1:**
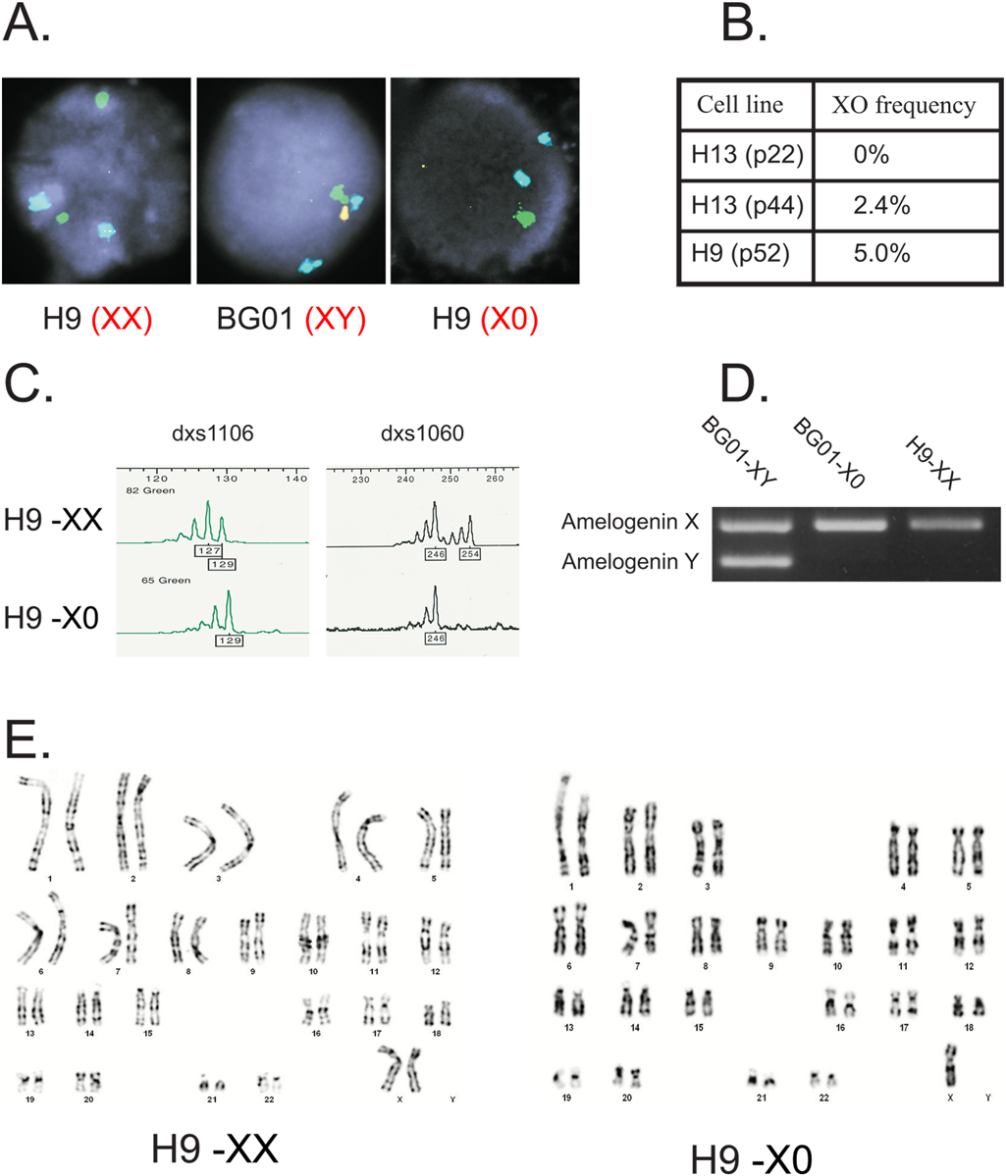
Isolation of XO clones from XX and XY HESCs. A. The frequency of X or Y chromosome loss in HESCs were estimated by FISH analysis. Shown are results for X chromosome (Green) Y chromosome (Yellow) and chromosome 17 (Blue), for H9 (XX) cells, BGO1 (XY cells) and a clone of H9 that has lost one of its X chromosomes. B. Summary of the % of XO cells analyzed by FISH. The analyzed samples were either male H13 cells passage 22 and passage 44, or female H9 cells, passage 52. In each experiment 200 cells were analyzed. C. Analysis of two polymorphic markers (DXS1106 and DXS1060) on the X chromosome that are heterozygous in H9 cells. XO cells retain only one of the markers. D. PCR products of primers that distinguish between Amelogenin X and Amelogenin Y genes. XY cells (BG01) have two products whereas XX cells (H9) have only one product. XY cells that have lost the Y chromosome amplify only one band. E. Karyotype analysis of XX and XO cells. Note that in the XO cells only one X chromosome is shown.

Our aim was to isolate 45,XO human embryonic stem cells (HESCs) and study their differentiation in culture. In order to estimate the frequency of spontaneous X or Y chromosome loss (in XX and XY cell lines, respectively) during *in vitro* culture of HESCs, we performed FISH analysis with probes against X, Y and the autosomal 17 chromosome (as a control) see Figure 1A. The results of the FISH analysis of 200 cells for two HESC lines are summarized in a table in Figure 1B. Overall we observed 2.4%–5% of loss of sex chromosomes. In H13 HESC line at passage 22 we could not detect any cells which had lost the Y chromosome, while in passage 44 of the same line 2.4% of the cells had lost the Y chromosome resulting in XO cells. This data suggesting a correlation between the passage number, and the frequency of the loss of sex chromosomes are in agreement with a previous study which reports a frequency of 2% in the loss of the Y chromosome in mouse ESCs [21]. Based on the relatively high frequency of X and Y chromosome loss as determined by the FISH analysis we have utilized a simple approach in order to isolate HESC clones that had lost one of their X chromosomes (in the case of female line) or their Y chromosome (in the case of male line). In order to ensure that each clone was originated from a single cell, the HESCs were transfected with a plasmid carrying the neo-resistance gene. Every colony that survived G418 selection was considered to be a “single cell clone” and was plated in a separate well. DNA was isolated from each clone and analyzed by PCR to allow the identification of X or Y chromosome loss. In order to detect 45,XO clones that originate from 46,XX cells we examined several heterozygous polymorphic microsattalites on the X chromosome. The loss of the X chromosome results in the disappearance of one of these polymorphic markers see Figure 1C.

In order to detect 45,XO clones that originate from 46,XY cells we utilized DNA primers for the Amelogenin gene. These primers distinguish between Amelogenin X and its Amelogenin Y homolog. Clones that lost their Y chromosome show only the Amelogenin X product, see Figure 1D. Karyotype analysis of one of the XO clones and of its original WT XX HESC line (H9) is shown in Figure 1E. About 3% (5/166) of the XX and 1.5% (1/67) of the XY clones were found to become XO clones. These results are in accordance with our FISH results.

### Searching of imprinted genes on the X chromosome

**Figure 2:**
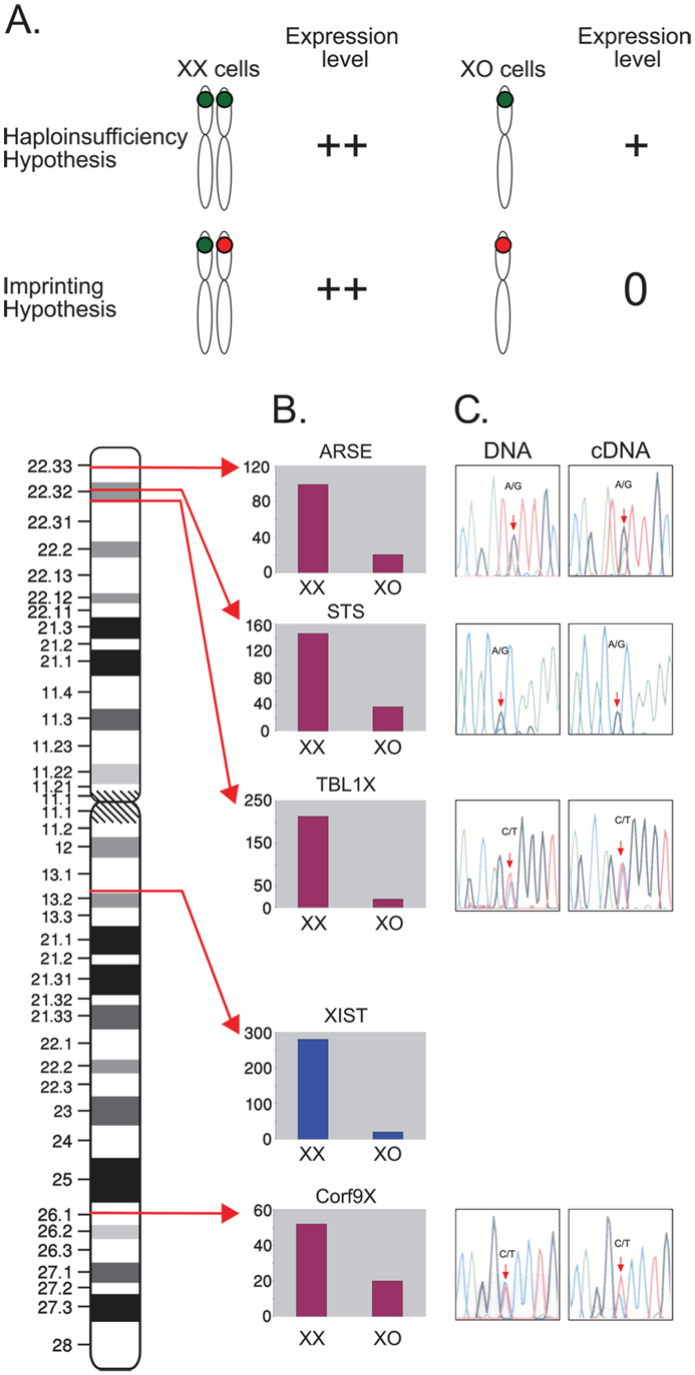
Searching for monoallelic expression in X chromosome. A. A scheme demonstrating the differences between the haploinsufficiency hypothesis and the imprinting hypothesis to explain the phenotype in X monosomy. Green dots represent expressed alleles and red dots represent silenced alleles. Note that according to the imprinted genes hypothesis there is no expression of the remaining allele in XO cells. B. Expression levels of several genes on the X chromosome whose expression is much higher in WT than in XO HESCs. The expression levels of STS, XIST and CXorf9 are shown in 30 d EBs and of ARSE and TBL1X in undifferentiated cells. XX – average of three microarray analyses of diploid HESCs. XO – average of two XO clones that have lost the same X chromosome. C. Searching for monoallelic expression in the candidate genes by SNP analysis at the DNA and cDNA levels.

The phenotype of Turner's syndrome was suggested to result from haploinsufficiency of pseudoautosomal gene/s on the X chromosome. It was also suggested that imprinted gene/s on the X chromosome are also involved in the phenotype (Figure 2A). Thus, we decided to search for developmentally regulated imprinted genes on the X chromosome in HESCs and in differentiated cells derived from HESCs. In order to search for this type of gene we set two criteria: 1. The gene has to be either pseudoautosomal or escape X inactivation, 2. The gene has to be expressed in XX cells but not expressed (or expressed at a very low level) in XO cells. 37 genes that escape X inactivation were analyzed in our microarray (Affymetyrix). Out of these genes, 21 genes are expressed in undifferentiated HESCs or in HESCs that underwent differentiation. From these genes we have identified only three X linked genes – ARSE (NM_000047), STS (NM_000351) and TBL1X (NM_005647) that fulfill the above criteria (see Figure 2). These genes escape X inactivation [22], [23] and are expressed in XX cells but not in XO cells. CXorf9 (NM_018990) is an example of a gene that is expressed in XX cells but not in XO cells, but undergo normal X inactivation; this gene was not expected to be an imprinted gene and served as control. XSIT (NR_001564) should be activated only in cells that have two or more X chromosomes and therefore it was expected that it will not be expressed at all in XO cells. In order to examine whether ARSE, STS and TBL1X are indeed expressed in a monoallelic manner, we looked for heterozygous SNPs in the DNA and mRNA molecules in these genes. In imprinted genes, it is expected that only one allele is expressed at the mRNA level, and thus the heterozygous SNP will appear as homozygous when sequencing the cDNA. None of the three candidate genes showed monoallelic expression, see Figure 2. Thus, according to our criteria, we could not identify imprinted genes on chromosome X.

### Cell specific effects of X chromosome loss

**Figure 3:**
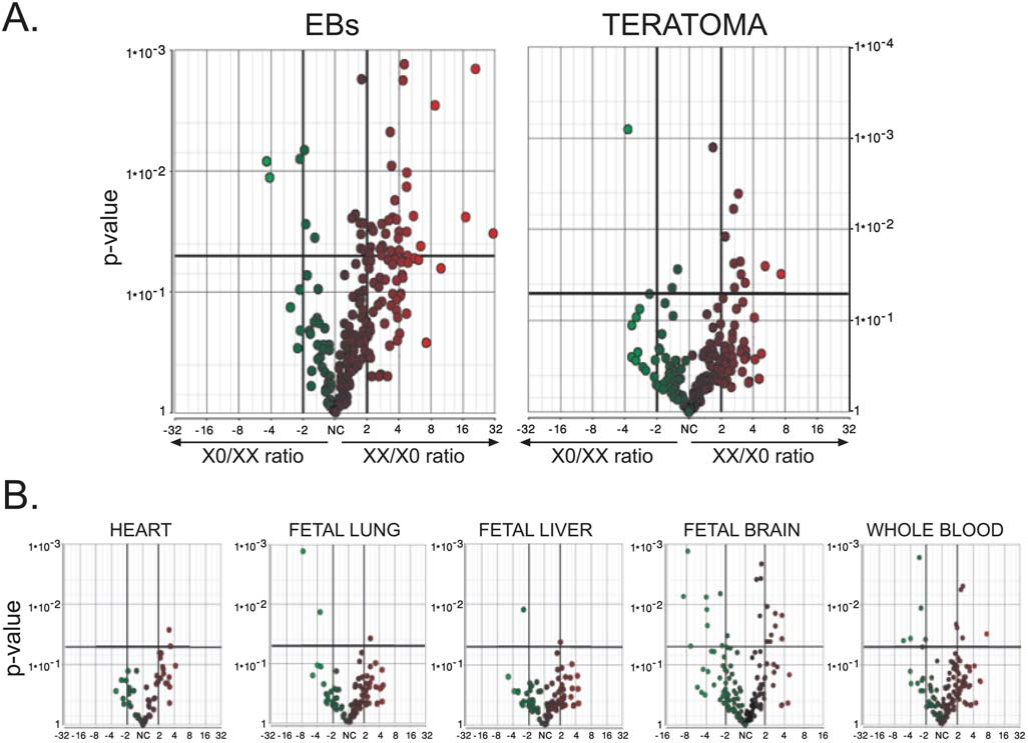
Comparison of gene expression in WT and in XO cells upon *in vitro* and *in vivo* differentiation. Gene expression levels were determined by U133A DNA microarray. Every dot represents one probe in the DNA microarray. In each plot the X axis represents fold induction in the expression levels of WT cells over XO cells (right side of the scale) and of Xo cells over WT cells (left side of the scale). The Y axis represents the P-value for each gene. For more details on the bioinformatic analysis see Materials and Methods. A. Analysis of genes specific to the placenta. B. Analysis of genes specific to the heart, fetal lung, fetal liver, fetal brain and whole blood. EBs – *in vitro* differentiation of HESCs. Teratoma – *in vivo* differentiation of HESCs. Solid vertical lines represents>2 fold higher level of expression solid horizontal line represent P-value = 0.05.

The fact that 99% of XO fetuses are lost during the first trimester [3] suggests that the early lethality of XO embryos results from a problem during human organogenesis. Human embryonic stem cells can differentiate *in vitro* and *in vivo* into extra-embryonic cells as well as into cells of the three embryonic germ layers [12]. Thus, we have decided to compare the differentiation of WT HESCs to that of XO HESCs. Using the Affymetrix U133A micryoarray we have studied both *in vitro* differentiation [9] and *in vivo* differentiation (teratomas) of HESCs. Since we wished to identify the differentiated cell types that are affected in XO cells, we divided the gene expression microarray data into lists of genes enriched in different tissues, and compared the gene expression of WT and XO differentiated HESCs. The assignment of genes into different tissues was performed according to the Gene Expression Atlas analysis [24]. Genes were determined to be highly enriched in a specific tissue if the ratio of their expression in the specific tissue was 30–50 fold higher than the median of its expression among all of the tissues in the analysis (note that this criterion doesn't confirm tissue specificity but rather, enrichment in a specific tissue). A list of the enriched genes is in supplementary Table S1. Using the above list we have compared the expression levels in XX and XO HESCs in several different tissues. The only tissue where many genes were expressed at higher levels in WT cells as compared to XO cells both upon *in vivo* and *in vitro* differentiation was the placenta see Figure 3A. (see supplementary Table S2). This is in contrast to other tissue-enriched genes that were examined, see Figure 3B. Several genes of the fetal brain were also expressed at higher levels in WT cells, however, in the fetal brain there were also genes that were expressed at higher level in the XO cells.

**Figure 4:**
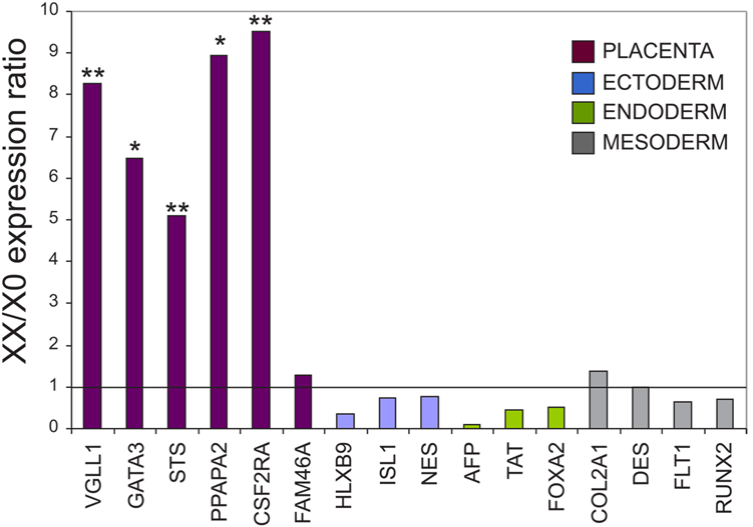
Confirmation of the DNA microarray data by qRT-PCR. Relative expression levels of WT vs. XO cells for several genes enriched in either the placenta, or in tissues that correspond to the ectoderm, endoderm or mesoderm embryonic germ layers. The genes were analyzed by qRT-PCR. The solid line represents equal expression in WT and XO cells. * represents p value<0.05 and ** represents p value<0.01.

The microarray results were confirmed by qRTPCR as shown in Figure 4. Except for one gene (FAM46A), all placental genes were expressed at least 5 fold higher in the WT cells as compared to the XO cells. On the other hand, we did not observe significant differences in the expression levels of genes representing the three embryonic germ layers between WT and XO cells.

## Discussion

One of the most important uses of HESCs is modeling for human developmental genetic diseases [16], [17], [25]. There are many developmental diseases that cannot be studied by animal models since the mutant animal does not recapitulate the human phenotype. In addition, many of these developmental disorders cannot be studied by cells from patients since these cells are terminally differentiated. In these cases, HESCs can be used as a complementary model system that overcomes these impediments.

In order to model genetic diseases by HESCs, one must have HESCs carrying a specific mutation. Several ways have been suggested for this purpose [25], [26]. Previously we have created a model for Lesch-Nyhan disease by gene targeting of the HPRT1 gene [16]. Later, we have established a model for Fragile X syndrome by the derivation of a new HESC line from a blastocyst identified by preimplantation genetic diagnosis (PGD) as a carrier of the Fragile X syndrome mutation [17]. In the current work we have used HESCs that have spontaneously lost one of their sex chromosomes in order to study the basis for the early lethality in 45,XO embryos. Approximately 3% of the pregnancy start with 45,XO embryos, however up to 99% of these embryos are spontaneously miscarried [3], most of them during the first trimester [4]. We have estimated that the frequency of spontaneous loss of X or Y chromosomes is 2–5%. The most frequent chromosomal aberration in HESCs are trisomies of 12, 17, X chromosome [27]. Although a few other chromosomal aberrations were also reported [27], the loss of either X or Y chromosomes during the culture of HESCs has not been previously described, probably since it is too rare to be found in regular karyotypic analysis. However the ratio of Y chromosome loss that we observed in HESCs is similar to the ratio reported by Eggan et al. in mouse ESCs [21]. In addition, the frequency of sex chromosome loss was higher in higher passage than in lower passage cells as demonstrated for other chromosomal aberrations in HESCs [28], [29].

Recently, in a milestone experiment, Takahashi and Yamanaka [30] have shown reprogramming of mouse somatic cells into pluripotent, embryonic-like cells by the ectopic expression of only a four genes. A few reports that followed the initial experiment have demonstrated reprogramming of human adult cells into induced pluripotent stem cells (iPS) [31]–[33]. Reprogramming of human somatic cells from affected individuals may enable us to generate models of human genetic diseases from cells of virtually any disorder, and thus open a new window of opportunity for modeling human genetic diseases [31]–[33]. However, generation of iPS from Turner's syndrome patients might not advance our understanding on the early lethality, since the patients are the exceptional cases that survived to term. Thus, one may need to study HESCs (and not iPS cells) with X monosomy to be able to analyze the developmental phenotype.

Our XO clones were established from the H9 HESC line. It has been shown in our lab that this line undergoes X inactivation only upon differentiation [34]. As expected, our XO clones didn't express XIST upon differentiation and the remaining X chromosome was active. Thus, we were able to isolate XO clones that have lost one of their X chromosomes.

In order to study the effect of the XO karyotype, we compared the transcriptional profiles of WT and XO clones. Among the different tissues that we studied, the only tissue that showed a significance upregulation upon both *in vivo* and *in vitro* differentiation was the placenta. Thus, we suggest that at least one of the reasons for the early lethality of XO embryos is abnormal placental development. The differences that we observed in the fetal brain may point to a more complex effect of XO monosomy, where some genes underwent down regulation upon X chromosome loss while others were upregulated. The fact that these results are reproducible in different XO clones suggests that the reason for the abnormal placental differentiation is indeed the lost of the X chromosome and not other genetic instabilities events.

In a detailed study of 160 spontaneous aborted 45,XO embryos as well as data from the literature Canki et al. [35] divided the aborted fetuses into 4 groups according to their morphology. Two out of the four groups which contain 70% of the total number of samples demonstrate early lethality in XO embryos even before mature embryonic tissues are visible. Our results which point to aberrant placental differentiation, may explain the morphological observation that many embryos had ruptured sacs. Based on an extensive study of the gene expression patterns in human placenta [36], we conclude that the down regulation in the expression of the placental genes in the XO clones, includes genes of the amnion, chorion, and villus parenchyma. Thus, we propose that the effect of the monosomy X on the placental differentiation is very early and therefore, has a general influence on the various components of the placenta. Mouse ES cells do not readily differentiate into the trophectoderm. In contrast, BMP4 was shown to efficiently direct differentiation of hESCs into the trophoblast [37]. In our system we have examined the differentiation of hESCs into placental cells *in vitro*, in mature embryoid bodies, and *in vivo*, in teratomas. Since in these two systems many different cell types exist, the various cell lineages served as internal controls and allow us to document a specific effect on the placental genes in the XO cells.

One possible mechanism that has been suggested to explain the etiology of early lethality in XO embryos is the presence of imprinted genes on the X chromosmes [5]. Using our XO HESCs we were not able to identify any imprinted genes on the X chromosome. We cannot rule out the possibility that there are X-linked imprinted genes which do not expressed in HESCs or upon *in-vitro* and *in-vivo* differentiation, and thus were not identified by our method. However, the fact that most of the XO embryos die during the first trimester, implies that an early and general developmental process is affected by the lost of the X chromosome. Spontaneous differentiation of HESCs can mimic the gastrulation and the formation of the three embryonic germ layers as well as the extraembryonic cells [13], [14]. Therefore, we assume that our system is sensitive enough to identify imprinted genes that have such a dramatic effect embryonic development, and that the fact that we didn't find imprinted X-linked genes, suggests that the early lethality in XO embryos is not caused by null expression of this type of genes.

The most common explanation for the early lethality of XO embryos is a haploinsufficiency effect of pseudoautosomal genes on the X chromosome. Therefore, we suggest that pseudoautosomal genes on the X chromosome have an important role in placental development. Two X linked genes that escape X inactivation are highly enriched in the placenta – STS and CSF2RA (NM_006140). Based on 39,XO mouse model, the STS gene has been suggested to play a role in the phenotype of Turner's syndrome patients [38]. However the human STS gene has no active homologue on the Y chromosome [39], and therefore it is not likely that haploinsufficiency of this gene causes the placental phenotype. The CSF2RA (colony-stimulating factor 2 receptor alpha) gene is an excellent candidate gene to explain the placental phenotype. This gene encodes the alpha subunit of the receptor of granulocyte-macrophage colony-stimulating factor (GMCSF NM_000758) which is essential for normal placental development [40]. Therefore, it is possible that haploissuficiency of the receptor (CSF2RA) will result in abnormal placental differentiation. Theoretically, the expression level of pseudoautosomal gene is supposed to be 2 fold higher in XX cells than in XO cells, however according to the qRTPCR results the CSF2RA gene is expressed 9.5 fold higher in the XX cells than in the XO cells (see Figure 4). We assume that the low expression levels in the XO cells is as a result of the unsuccessful placental differentiation of the XO HESCs. The outcome of the haploinsufficiency of CSF2RA (which result from relatively minor decrease in the expression level of the gene) may cause a general defect in placental differentiation and down regulation of many placental genes included CSF2RA itself. The fact that XO mice are fertile and healthy suggests that the genes involved in the human XO phenotypes (included the early lethality) are either autosomal or undergo X inactivation in mice and thus there is no haploinsufficiency effect for this gene in XO mice [11]. Indeed, the CSF2RA gene is autosomal in mice and resides on chromosome 19.

In conclusion, we suggest that the embryonic lethality in XO embryos results from a defect in placental differentiation. The defect may be a consequence of haploinsufficiency in the expression of a placental gene that resides on the X chromosome specifically in humans.

## Supporting Information

Table S1 Tissues specific genes according to GNF
(0.21 MB XLS)

Table S2 Placental genes higher in XX cells than in XO cells
(0.03 MB XLS)

Table S3 (0.03 MB DOC)

Table S4 (0.04 MB DOC)

Table S5 (0.04 MB DOC)

Table S6 (0.04 MB DOC)
