# Supplementary material for: Studying Early Lethality of 45,XO (Turner's Syndrome) Embryos Using Human Embryonic Stem Cells
Source: PLoS One. 2009 Jan 12;4(1):e4175. doi: 10.1371/journal.pone.0004175 (PMC2613558; doi:10.1371/journal.pone.0004175)
Supplement: Table S3 — (0.03 MB DOC) [file pone.0004175.s003.doc]

**Supplementary Table 3 - Primers for PCR for Amelogenin X,Y**

| **Gene** | **5’ primer** | **3’ primer (common)** | **size**  **(bp)** |
| --- | --- | --- | --- |
| **AMELOGENIN X**  (NM_001142) | CAGCTTCCCAGTTTAAGCTCT | GCCCAAAGTTAGTAATTTTACCT | 329 |
| **AMELOGENIN Y**  (NM_001143) | TCTCCTATACCACTTAGTCACT |  | 235 |
