# Supplementary material for: Studying Early Lethality of 45,XO (Turner's Syndrome) Embryos Using Human Embryonic Stem Cells
Source: PLoS One. 2009 Jan 12;4(1):e4175. doi: 10.1371/journal.pone.0004175 (PMC2613558; doi:10.1371/journal.pone.0004175)
Supplement: Table S4 — (0.04 MB DOC) [file pone.0004175.s004.doc]

**Supplementary Table 4 - Primers for qRT-PCR for placental genes:**

| **Gene** | **5’ primer** | **3’ primer (common)** | **size**  **(bp)** |
| --- | --- | --- | --- |
| **VGLL1** | CACCTACTTCCAAGGGGACA | AACGCCACTGATTTGGAGAC | 155 |
| **GATA3** | AAGGCAGGGAGTGTGTGAAC | TCCTCCAGAGTGTGGTTGTG | 213 |
| **STS** | ATTGGAGATCCTGGGTGCTA | GACCAAGATGCCATTCCTGA | 175 |
| **PAPPA2** | ACGACTTTGACGACGGAGAC | GCACTGAGCTGGCAAAGTAG | 168 |
| **CSF2RA** | CGCTGCTCAGAATTTCTCCT | CCACATGGGTTCCTGAGTCT | 173 |
| **FAM46A** | CTGGACTGCCTGTTGGACTT | CCACATTTTTGCCACTGTTG | 158 |

Endogenous control for qRT-PCR

| **Gene** | **Taqman probe** |
| --- | --- |
| UBC  (M26880) | Hs00824723_m1 |
| GAPDH  (AK026525) | Hs99999905_m1 |
