# Supplementary material for: Studying Early Lethality of 45,XO (Turner's Syndrome) Embryos Using Human Embryonic Stem Cells
Source: PLoS One. 2009 Jan 12;4(1):e4175. doi: 10.1371/journal.pone.0004175 (PMC2613558; doi:10.1371/journal.pone.0004175)
Supplement: Table S5 — (0.04 MB DOC) [file pone.0004175.s005.doc]

**Supplementary Table 5 - TaqMan probes form TaqMan Low Density Array HUMAN STEM CELL:**

| **Gene** | **Taqman probe** | **Gene** | **Taqman probe** |
| --- | --- | --- | --- |
| HLXB9  (NP_005506) | Hs00232128_m1 | PECAM1  (NM_000442) | Hs00169777_m1 |
| AFP  (NM_001134) | Hs00173490_m1 | CDH5  (NM_001795) | Hs00174344_m1 |
| IL6ST  (NM_002184) | Hs00174360_m1 | COL2A1  (NM_001844) | Hs00156568_m1 |
| NES  (NM_006617) | Hs00707120_s1 | FOXA2  (NM_021784) | Hs00232764_m1 |
| COL1A1  (NM_000088) | Hs00164004_m1 | RUNX2  (NM_004348) | Hs00231692_m1 |
| DES  (NM_001927) | Hs00157258_m1 | TAT  (NM_000353) | Hs00356930_m1 |
| FLT1  (NM_002019) | Hs00176573_m1 | PECAM1  (NM_000442) | Hs00169777_m1 |

Endogenous control for TaqMan Low Density Array

| **Gene** | **Taqman probe** |
| --- | --- |
| RAF1  (NM_002880) | Hs00234119_m1 |
| CTNNB1  (NM_001904) | Hs00170025_m1 |
| EEF1A1  (NM_001402) | Hs_00742749_s1 |
