# Supplementary material for: Studying Early Lethality of 45,XO (Turner's Syndrome) Embryos Using Human Embryonic Stem Cells
Source: PLoS One. 2009 Jan 12;4(1):e4175. doi: 10.1371/journal.pone.0004175 (PMC2613558; doi:10.1371/journal.pone.0004175)
Supplement: Table S6 — (0.04 MB DOC) [file pone.0004175.s006.doc]

**Supplementary Table 6 - Primers for SNPs analysis (PCR and RT-PCR):**

| **Gene** | **SNP** |  | **5’ primer** | **3’ primer** | **size**  **(bp)** |
| --- | --- | --- | --- | --- | --- |
| **TBL1X** | rs.2106705 | gDNA | CAGAAGGGCTGAGGAAGATG | GTGAATTGCTTGCGACACAT | 620 |
|  |  | cDNA | CTTCGACAAGTGCGTCCATA | AAGTTCCACAGAAGCCCTGA | 2182 |
|  |  | cDNA (nested) | CAGAAGGGCTGAGGAAGATG | AAGTTCCACAGAAGCCCTGA | 173 |
| **ARSE** | rs. 11222 | gDNA | AGCCCGTGTTCTATCAGGTG | GCCAGCTGGTTTGTTTCAAT | 249 |
|  |  | cDNA | GACAGCCCAACACTCAGACC | CATCTTCCCTAAGGCACCAG | 431 |
| **STS** | rs. 1131289 | gDNA | TGGAAGCCCACTTCTCAGAT | CTGTGCGCTAATACCCTGCT | 200 |
|  |  | cDNA | AGCCCACTAGCAACATGGAC | CACTCTCGGATCATGGAGGT | 3579 |
|  |  | cDNA (nested) | TGGAAGCCCACTTCTCAGAT | CACTCTCGGATCATGGAGGT | 386 |
| **CXorf9** | rs. 859577 | gDNA | ACTGAGGAGCAGCTGCAAG | AGCTGAGCCTGGAACCTGTA | 220 |
|  |  | cDNA | CTGCGAGAAACACACCTCAA | AGCTGAGCCTGGAACCTGTA | 460 |
